# FGF22 deletion causes hidden hearing loss by affecting the function of inner hair cell ribbon synapses

**DOI:** 10.3389/fnmol.2022.922665

**Published:** 2022-07-28

**Authors:** Shule Hou, Jifang Zhang, Yan Wu, Chen Junmin, Huang Yuyu, Baihui He, Yan Yang, Yuren Hong, Jiarui Chen, Jun Yang, Shuna Li

**Affiliations:** ^1^Department of Otorhinolaryngology-Head and Neck Surgery, Xinhua Hospital, School of Medicine, Shanghai Jiao Tong University, Shanghai, China; ^2^Ear Institute, School of Medicine, Shanghai Jiao Tong University, Shanghai, China; ^3^Shanghai Key Laboratory of Translational Medicine on Ear and Nose Diseases, Shanghai, China; ^4^Liaoning Medical Device Test Institute, Shenyang, China; ^5^Laboratory of Electron Microscope Center, Shanghai Medical College, Fudan University, Shanghai, China; ^6^Department of Otorhinolaryngology-Head and Neck Surgery, Shanghai Children’s Hospital, Shanghai Jiao Tong University, Shanghai, China

**Keywords:** FGF22, ribbon synapse, hidden hearing loss, SNAP-25, Gipc3, MEF2D

## Abstract

Ribbon synapses are important structures in transmitting auditory signals from the inner hair cells (IHCs) to their corresponding spiral ganglion neurons (SGNs). Over the last few decades, deafness has been primarily attributed to the deterioration of cochlear hair cells rather than ribbon synapses. Hearing dysfunction that cannot be detected by the hearing threshold is defined as hidden hearing loss (HHL). The relationship between ribbon synapses and FGF22 deletion remains unknown. In this study, we used a 6-week-old FGF22 knockout mice model (*Fgf22*^–/–^) and mainly focused on alteration in ribbon synapses by applying the auditory brainstem response (ABR) test, the immunofluorescence staining, the patch-clamp recording, and quantitative real-time PCR. In *Fgf22*^–/–^ mice, we found the decreased amplitude of ABR wave I, the reduced vesicles of ribbon synapses, and the decreased efficiency of exocytosis, which was suggested by a decrease in the capacitance change. Quantitative real-time PCR revealed that *Fgf22*^–^*^/^*^–^ led to dysfunction in ribbon synapses by downregulating SNAP-25 and Gipc3 and upregulating MEF2D expression, which was important for the maintenance of ribbon synapses’ function. Our research concluded that FGF22 deletion caused HHL by affecting the function of IHC ribbon synapses and may offer a novel therapeutic target to meet an ever-growing demand for deafness treatment.

## Introduction

The mammalian cochlea consists of outer hair cells (OHCs) and inner hair cells (IHCs). OHCs amplify sound, thereby increasing the hearing sensitivity of the mammalian inner ear. In contrast, IHCs are responsible for capturing acoustic signals and delivering auditory information to the central nervous system. In mammals, the loss of hair cells cannot be spontaneously replaced, but the functional alteration can be rescued before hair cell loss (Jan et al., 2013; Li et al., 2016; Ni et al., 2016; Waqas et al., 2016; Wu et al., 2016; Chen et al., 2017).

Ribbon synapses are important structures in transmitting auditory signals from the IHCs to their corresponding spiral ganglion neurons (SGNs). Ribbon synapses, which are located at the base of the IHCs, contain presynaptic RIBEYE and postsynaptic glutamate receptors. For the signals to be transmitted accurately, the presynaptic active domain needs to quickly and precisely release glutamate-filled synaptic vesicles (SVs) (Nouvian et al., 2006).

The fibroblast growth factor (FGF) family comprises 22 structurally related molecules that can be grouped into seven subfamilies based on their similarities and fibroblast growth factor receptor (FGFR) binding (Itoh and Ornitz, 2004). For example, FGFs 7, 10, and 22 are similar in sequence, and they all activate FGFR2b (Zhang et al., 2006).

Numerous phenotypic similarities in FGF signaling between mice and humans have been illustrated in the branching morphogenesis of the lung epithelium (Goto et al., 2014), heart development (Itoh et al., 2016), skeletal growth (Teven et al., 2014), muscle regeneration (Yablonka-Reuveni et al., 2015), angiogenesis and lymphangiogenesis (Yu P. et al., 2017), kidney development (Carev et al., 2008), neurogenesis and neurodegeneration (Woodbury and Ikezu, 2014), craniofacial suture ossification (Prochazkova et al., 2018), and ear development (Raft and Groves, 2015). Due to conserved developmental functions and genome accessibility, the mouse is a superb model to study the mechanisms of FGF signaling *in vivo*. Consequently, many experiments concerning FGFs in the auditory system have been performed in mice as well as in other animals. Previously, FGF signaling was reported to play an important role in otic placode specification (Wright et al., 2015), cochlear development (Ebeid and Huh, 2017), neuromast hair cell regeneration (Lee et al., 2016), inner ear hair cell differentiation (Leger and Brand, 2002; Haque et al., 2016; Ratzan et al., 2020), and middle ear formation (Lysaght et al., 2014).

FGF22, a member of the FGF family, plays an intermediary role in synapse reconstruction (Jacobi et al., 2015) and axon rehabilitation (Zhu et al., 2020). As a presynaptic organizer originating from a postsynaptic cell in mammals, FGF22 is vital for excitatory synapse formation in the hippocampus (Umemori et al., 2004; Terauchi et al., 2015). It also contributes to neural growth in the dentate gyrus (Terauchi et al., 2017). We previously demonstrated that FGF22 could protect against hearing loss resulting from gentamycin ototoxicity and generate ribbon synapses by suppressing myocyte enhancer factor 2D (MEF2D) (Li et al., 2020). However, how ribbon synapses are related to FGF22 deletion remains unclear.

In this study, an FGF22 knockout mice model (*Fgf22*^–/–^) was utilized. Wild-type mice (*Fgf22*^+/+^) served as controls. Hearing thresholds were unchanged, but a decrease in wave I amplitude and synaptic defects was found, suggesting that Fgf22−/− mice suffer from hidden hearing loss (HHL). A set of experiments, such as the ABR test, the immunofluorescence staining, and the patch-clamp recording, were performed. Then, we measured the gene expression of synaptosome-associated protein 25 (SNAP-25), GIPC PDZ domain-containing family member 3 (Gipc3), and MEF2D. In brief, we tried to clarify the relationship between FGF22 and HHL.

## Materials and methods

### Animals

All animal experiments were in accordance with the guidelines approved by the experimental animal care institution of Shanghai Jiao Tong University School of Medicine. The approval number is XHEC-F-2021-065.

In the study, the mice (C57BL/6 background, 6-week-old) were obtained from the Harvard Children’s Hospital (Terauchi et al., 2010). Then *Fgf22*^–/–^ mice were derived from a heterozygous mating scheme. Wild-type littermates of the same age were used as controls. The mice were housed in the group on a schedule of 12 h of light and 12 h of darkness, and they had free access to food and water. We inspected the ears of the mice, and those with infections in the external auditory canal and middle ear were excluded.

### Genotyping

Deoxyribonucleic acid (DNA) was extracted from mice tails using a TIANamp Genomic DNA Kit. Polymerase chain reaction (PCR) was then performed to determine the presence of *Fgf22* alleles; i.e., wild-type, *Fgf22* homozygous, and *Fgf22* heterozygous.

The primer sequences used for FGF22 genotyping are as follows: *Fgf22* GS (E) (5′-TGCCTGACCATCTACTCCTGTCTCC-3′), *Fgf22* GS (T, E) (5′-GAACCTACAGTCCACAGAGTAGACC-3′), and Neo (T) (5′-GGGCCAGCTCATTCCTCCCACTCAT-3′). After amplification, whole products were separated using 1% agarose gel electrophoresis. GeneRed (TIANGEN, China) was used to stain the gel, and the stained gel was viewed under ultraviolet light.

### Auditory brainstem response testing

Mice (*n* = 12) were weighed and anesthetized with intraperitoneal injections of 150 mg/kg ketamine and 6 mg/kg xylazine. ABR recordings were carried out in a soundproof chamber by placing the electrodes subcutaneously, and the TDT RZ6 system was used for testing. The recording electrode was placed at the vertex of the head, while the stimulating electrode was inserted into one mastoid process. The ground electrode was inserted into the contralateral thigh. The sensing resistance was <1 kΩ.

The ABR test was carried out using BiosigRZ software. The sound stimulation output at 4–32 KHz was presented at a rate of 20 pips per second, lasted for 3 ms, and was overlaid 400 times. The sound was delivered to the ears *via* a loudspeaker placed 10 cm from the pinna. The stimulating intensities ranged from 10 to 90 dB sound pressure levels (SPLs). The hearing threshold, defined as the lowest stimuli level triggering a replicable response, was measured using decrements from high to low intensity in steps of 5 dB SPL. Additionally, the ABR wave I amplitudes and peak latencies were identified manually (Song et al., 2006).

### Tissue preparation

Mice (*n* = 12) were quickly decapitated after anesthesia with 150 mg/kg ketamine and 6 mg/kg xylazine intraperitoneally. The temporal bones were taken off and immersed in fresh 4% paraformaldehyde (PFA). The walls of the auditory sac and footplates of the stapes were cleared. Then, the round and oval windows were opened, and a hole was drilled into the cochlea apex from where 4% PFA solution was perfused. After fixation, the basal membrane (middle turn) was gently separated from the cochlea for whole-mount staining.

### Immunofluorescence staining

For immunofluorescence staining, we used antibodies against rhodamine-phalloidin (1:200, Yeasen, #40734ES75), C-terminal binding protein-2 (CtBP2, 1:400, mouse IgG1, BD Bioscience, #612044^1^), glutamate receptor 2 (GluR2, 1:200, mouse IgG2a, Millipore, Cat #mab397^2^), and 4′, 6-diamidino-2-phenylindole (1:1,000, Sigma, United States, #D9542).

Whole-mount organs of Corti were permeabilized with 0.1% Triton X-100 for 40 min and then blocked with 10% normal goat serum for 30 min at 37°C. Subsequently, the tissue was incubated with the primary antibodies overnight at 4°C. After rinsing, the samples were incubated with their specific secondary antibodies: 1:500 of goat anti-mouse IgG1 (Invitrogen, Alexa Flour™ 568-conjugated, ref#A21134, RRID: AB_2535766) and goat anti-mouse IgG2a (Invitrogen, Alexa Flour™ 647-conjugated, ref#A21241, RRID: AB_2535810) at 37°C for 2 h. The samples were imaged using a laser confocal microscope (Leica SP8, Germany).

### 3D reconstruction using a laser confocal microscope

Whole-mount organs of Corti were imaged using a 60× water-immersion objective coupled to a laser confocal microscope (Zeiss LSM 880, Germany). The presynaptic (CtBP2) and postsynaptic structures (GluR2) were detected in the red and green channels, respectively. Each orange fluorescent dot (i.e., red and green merge) indicated one ribbon synapse. Continuous scans were carried out from top to bottom with a step size of 0.5 μm. After each layer was scanned, the signals from each layer were obtained, and then ribbon synapses were quantified *via* signal superposition.

### Transmission electron microscopy

Ribbon-attached vesicles were counted using transmission electron micrographs of complete serial sections encompassing the ribbon synapses.

The cochleae (*n* = 6) were fixed in 2.5% glutaraldehyde/0.1 M phosphate buffer and decalcified by immersion in a 10% ethylene diamine tetraacetic acid (EDTA) solution. Afterward, the specimens were postfixed in a 1% osmium acid solution for 2 h at room temperature. Following postfixation, the specimens were rinsed three times for 5 min each with 0.1 M butylene succinate. Next, the sections were dehydrated using gradients of ethanol and acetone solution. For embedment, the specimens were immersed in a 1:1 solution of acetone and Epon 812 for 2–4 h, followed by a 2:1 acetone:Epon 812 solution overnight. Then, they were immersed in Epon 812 for 5–8 h. The samples were subsequently inserted, while immersed in Epon 812, into an embedment plate in a 37°C incubator overnight; afterward, they were polymerized in a 60°C incubator for 36 h. Using an ultramicrotome, semithin sections (2.0 μm) were sliced serially to the central axis plane and collected onto glass slides. The sections were unfolded and dried on a 95°C heating plate; afterward, they were stained with a 1% toluidine blue for a few seconds. The standard plane of the cochlear axis was located under the microscope. Additionally, ultrathin sections (50–60 nm) were sliced using an ultramicrotome. The sections were stained with 2% uranyl acetate saturated alcohol solution for 15 min. After the first stain was completed, the tissues were stained with lead citrate for 15 min. Finally, the dual-stained sections were dewatered at room temperature overnight. IHC ribbon synapses were imaged using a transmission electron microscope (Philips CM-120) for analysis.

### Patch-clamp recording

The IHCs were oriented for the patch-clamp experiment using the AXON patch-clamp system. Recording pipettes were pulled from borosilicate glass (World Precision Instruments) to a resistance of 4–6 MΩ. The bath solution contained 130 mM sodium chloride, 1 mM magnesium chloride, 2.8 mM potassium chloride, 10 mM calcium chloride, 10 mM amphoteric HEPES buffer, and 10 mM D-glucose, pH adjusted to 7.20 with NaOH, and osmolarity adjusted to 300 mOsm with D-glucose. To increase the calcium current and capacitance jumps, 10 mM calcium chloride was added. The electrode liquid consisted of 135 mM Cs-methanesulfonate, 10 mM cesium chloride, 10 mM tetraethylammonium chloride, 10 mM amphoteric HEPES buffer, 2 mM calcium chelator EGTA, 0.5 mM Na-GTP, and 3 mM Mg-ATP, pH adjusted to 7.20 with CsOH and osmolarity adjusted to 290 mOsm with D-glucose. The jClamp software was used to acquire and analyze all data. Recordings were performed at room temperature (∼24°C). Cells were held at −80 mV (unless otherwise indicated). Traces were recorded immediately after the cell membrane was broken through at a giga-ohm (GΩ) seal, and the series resistance (Rs) and membrane capacitance (Cm) were corrected. Liquid junction potential (−9.3 mV) was corrected offline. Data with a leakage current of less than 30 pA were included in the statistics.

The current-voltage relationship, which displays how Ca^2+^ currents react to the voltage ramp, was quantified using a point-by-point calculation. The curve-fitting equation was as follows:


$$I\,\left(V\right)=\left(V-{\text{V}}_{\text{rev}}\right)\times \frac{{\text{G}}_{\text{max}}}{1\,e\,x\,p\,\left(-\left(V-{\text{V}}_{\text{half}}\right)/k\right)}$$

In the above equation, *V*_*rev*_ refers to the reversal membrane potential.

A constant double-sinusoidal wave (390.6 and 781.2 Hz, 20 mV) superposed on the holding potential (−80 mV) was used to record membrane capacitance at different depolarization times of 10, 30, 50, 100, and 200 ms. The membrane capacitance change (Δ*C*_*m*_) is the difference between the average capacitance before and after depolarization. The area from the beginning of the Ca^2+^ current to the end of the Ca^2+^ tail current was integrated using the Patchmaster software to obtain the calcium influx charge (*Q*_*Ca*_). The ratio of the capacitance change to the Ca^2+^ current charge (Δ*C*_*m*_/*Q*_*Ca*_) was used to quantify the efficiency of Ca^2+^ in triggering exocytosis.

### Quantitative real-time PCR

Freshly separated inner ears were collected from 6-week-old *Fgf22*^+/+^ (*n* = 6) and *Fgf22*^–/–^ (*n* = 6) mice. The apical-basal membrane was micro-dissected rapidly, and the samples were stored at −80°C until further treatment. All of the ribonucleic acids were extracted to synthesize the complementary DNA, which then acted as a template for the amplification. The PCR amplification was conducted using the primers which are listed in Table 1.

**TABLE 1:** Primers for quantitative real-time PCR.

Gene	Forward	Reverse
*SNAP-25*	5′-CGGATCCATGGCCGAGGA CGCAGACAT-3′	5′-TTAACCACTTCCC AGCATCTT-3′
*Gipc3*	5′-AGGATCGAGGGCTT CACCAAT-3′	5′-GCAACTTTTGCATG TCCACTTT-3′
*MEF2D*	5′-CGTTGGGAATGG CTATGTC-3′	5′-GAGGCCCTGGC TGAGTAA-3′
β*-actin*	5′-AAGGACTCCTATAGTG GGTGACGA-3′	5′-ATCTTCTCCATG TCGTCCCAG TTG-3′

β-actin was used as a reference.

The real-time PCR procedures were conducted as follows: 48°C for 30 min then 95°C for 5 min, followed by 40 cycles consisting of 94°C for 15 s, 60°C for 30 s, and 72° for 30 s. The reactions were replicated three times. The 2^–ΔCt^ method was employed to quantify messenger ribonucleic acid (mRNA) levels of the above-mentioned genes.

### Statistical analysis

**FIGURE 1:**
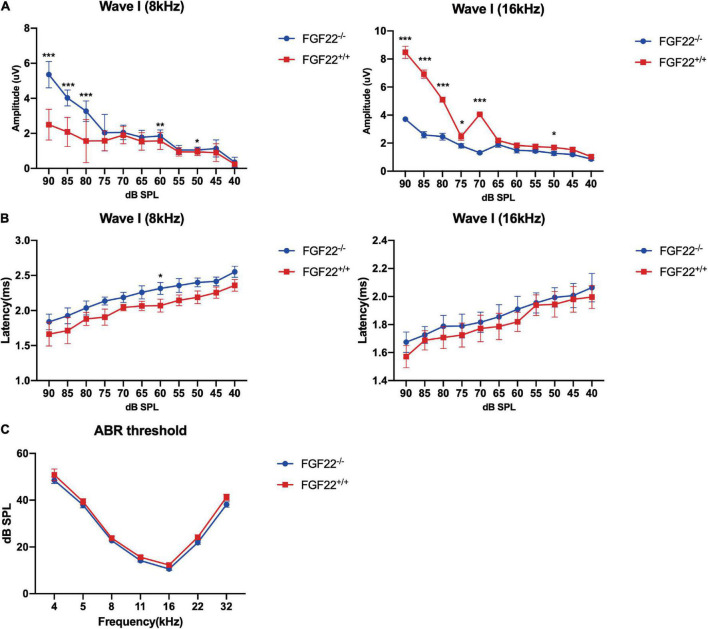
Auditory function parameters in the *Fgf22*^+/+^ and *Fgf22*^–/–^ groups. **(A)** Mean amplitudes of the ABR wave I in the *Fgf22*^+/+^ and *Fgf22*^–/–^ groups at 8 and 16 kHz. **(B)** Mean latency values of the ABR wave I in the *Fgf22*^+/+^ and *Fgf22*^–/–^ groups at 8 and 16 kHz. **(C)** Mean values of the ABR threshold in the *Fgf22*^+/+^ and *Fgf22*^–/–^ groups at 8 and 16 kHz. Data are expressed as the mean ± SD, statistical significance was assessed with two-way ANOVA followed by Bonferroni *post hoc* test, *P < 0.05, **P < 0.01, ***P < 0.001.

**FIGURE 2:**
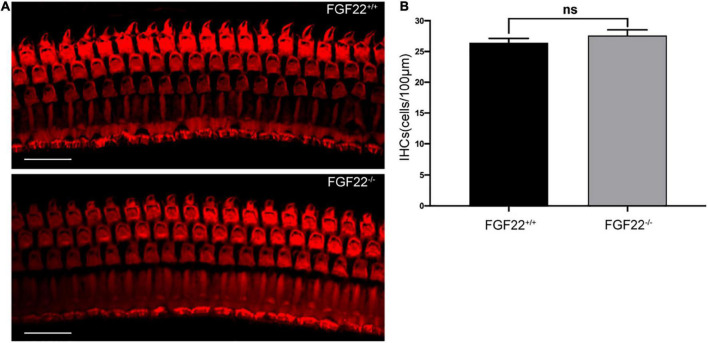
Whole-mount of the cochlea hair cells in *Fgf22*^+/+^ and *Fgf22*^–/–^ groups. **(A)** Isolated cochlea hair cells were immunostained with phalloidin (red). Scale bars: 20 μm. **(B)** The bar chart showed no difference in the hair cells between the *Fgf22*^+/+^ and *Fgf22*^–/–^ groups.

**FIGURE 3:**
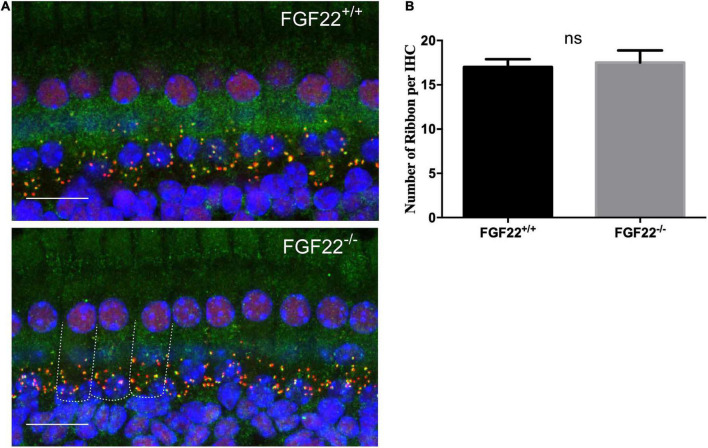
The number of synaptic ribbons in *Fgf22*^+/+^ and *Fgf22*^–/–^ mice. **(A)** Representative cochlear whole-mount preparation (*n* = 6, middle turn) images of CtBP2-labeled presynaptic ribbons (red) and GluR2-labeled postsynaptic glutamate receptors (green) from the *Fgf22*^+/+^ and *Fgf22*^–/–^ groups showing the overlapped puncta (yellow). Scale bars: 20 μm. **(B)** The bar chart showed the number of synaptic ribbons per IHC, which was similar in the *Fgf22*^+/+^ and *Fgf22*^–/–^ groups.

**FIGURE 4:**
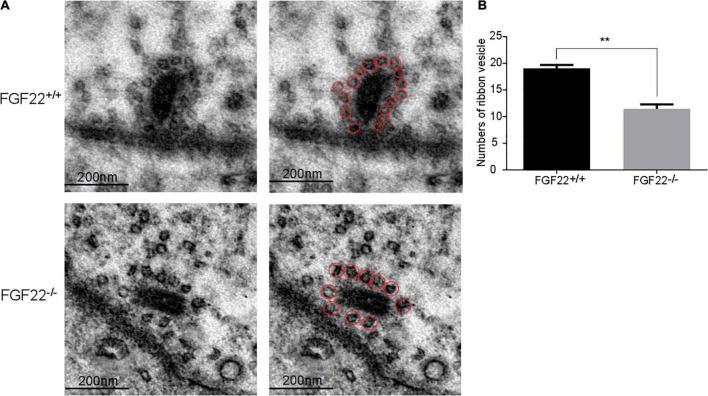
The number of synaptic vesicles in the *Fgf22*^+/+^ and *Fgf22*^–/–^ groups. **(A)** Representative images of synaptic vesicles in the *Fgf22*^+/+^ and *Fgf22*^–/–^ groups under a transmission electron microscopy (TEM). Scale bars: 200 nm. **(B)** The number of ribbon SVs was significantly reduced in *Fgf22*^–/–^ mice than in *Fgf22*^+/+^ mice. **P < 0.01 vs. the *Fgf22*^–/–^ group.

**FIGURE 5:**
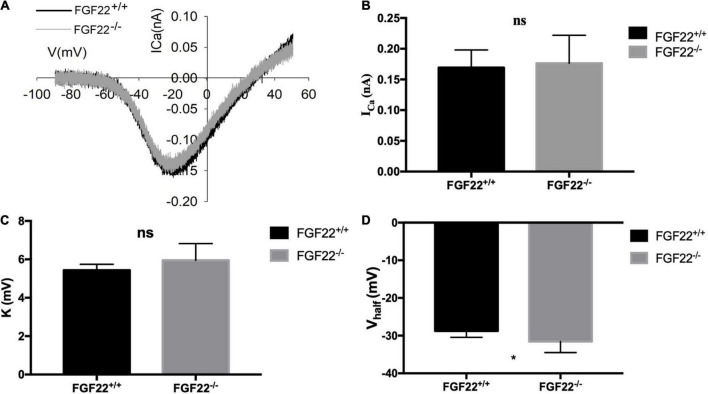
Changes in Ca^2+^ current in IHCs of the *Fgf22*^+/+^ and *Fgf22*^–/–^ groups. **(A)** Representative curves of the Ca^2+^ current in IHCs of the *Fgf22*^+/+^ (black) and *Fgf22*^–/–^ (gray) groups. The current response was induced by a voltage ramp from –80 to 60 mV and then the leak was subtracted. **(B,C)** No significance was found in the Ca^2+^ current amplitude (*I*_*Ca*_) and the slope factor (*k*) between the *Fgf22*^+/+^ and *Fgf22*^–/–^ groups. **(D)** IHCs from the *Fgf22*^–^*^/^*^–^ mice (–31.55 mV) have a more negative half-activation voltage (*V*_*half*_) than Fgf22^+/+^ mice (–28.76 mV). *P = 0.0301, which indicates significant differences with *P* < 0.05. Statistical significance was assessed with a two-way ANOVA followed by Bonferroni *post hoc* test.

**FIGURE 6:**
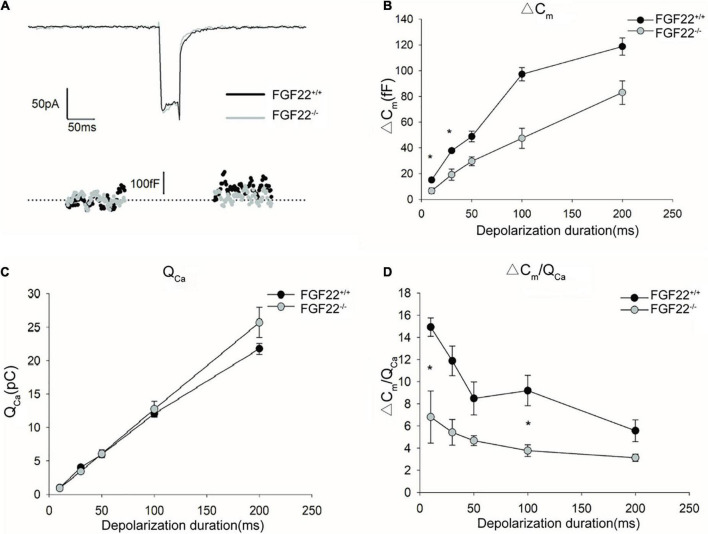
Changes in exocytosis in IHCs between the *Fgf22*^+/+^ and *Fgf22*^–/–^ groups. **(A)** Representative Ca^2+^ currents (*I*_*Ca*_) and the resulting capacitance jumps (Δ*C*_*m*_) recorded from IHCs between the *Fgf22*^+/+^ (black) and *Fgf22*^–/–^ (gray) groups. **(B,C)** Δ*C*_*m*_ and the Ca^2+^ charge (*Q*_*Ca*_) evoked by stimulations of different durations, from 10 to 200 ms. Δ*C*_*m*_ for stimulation of 10 and 30 ms was significantly reduced in the *Fgf22*^–/–^ group, *P = 0.0301, and *P* = 0.0251 < 0.05, respectively. **(D)** The Ca^2+^ efficiency of triggering exocytosis, assessed based on the ratio of Δ*C*_*m*_/*Q*_*Ca*_, was reduced significantly for stimulation of 10 and 100 ms in the *Fgf22*^–/–^ group. Data are expressed as the mean ± SD. *Indicates significant differences with *P* = 0.0476, and *P* = 0.0201 < 0.05, respectively.

**FIGURE 7:**
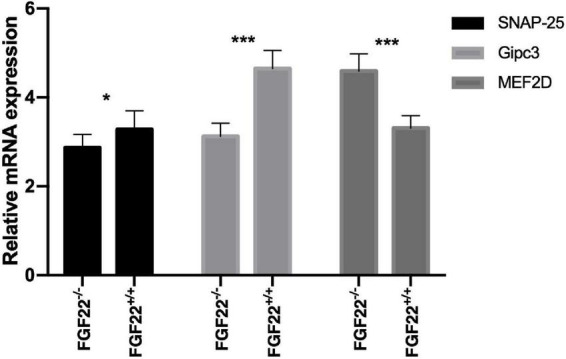
Quantitative real-time PCR analysis in the Corti’s organ of the *Fgf22*^+/+^ and *Fgf22*^–/–^ groups. *Fgf22*^–/–^ mice displayed downregulation of SNAP-25 and Gipc3 and upregulation of MEF2D. Data are expressed as the mean ± SD, and statistical significance was assessed with a two-way ANOVA followed by Bonferroni *post hoc* test, *P < 0.05, ***P < 0.001.

Data in Figures 1, 5–7 were analyzed by one-way analysis of variance using Graphpad Prism 8. Data in Figures 2–4 were analyzed by a two-tailed *t*-test using Excel 2019. A *P*-value of <0.05 indicates statistical significance. Statistical data were normally distributed and equal variances were assumed.

## Results

### The wave I amplitude decreased in *Fgf22*^–/–^ mice

Hidden hearing loss manifests as decreased wave I amplitude with normal hearing thresholds. We used the ABR to assess the hearing thresholds, amplitudes, and latencies of *Fgf22*^–/–^ mice. The wave I amplitude of *Fgf22*^–/–^ mice at 8 and 16 KHz was presented for the well-representative characteristics in hearing conditions, and it was significantly reduced compared to *Fgf22*^+/+^ mice (Figure 1A), while no significant difference in wave I latency or hearing threshold was observed between the two groups (Figures 1B,C).

### The number of inner hair cells was unchanged in *Fgf22*^–/–^ mice

The basal membranes obtained from *FGF22*^–/–^ mice were processed, and the isolated hair cells were immunostained with phalloidin (red). The images showed that no difference was found in the IHCs between *Fgf22*^–/–^ and *Fgf22*^+/+^ mice (27.60 ± 1.855 in *FGF22*^–/–^ mice vs. 26.40 ± 1.497 in *FGF22*^+/+^ mice; Figure 2). Most hearing impairments are closely associated with hair cell loss. And from this result, it does not appear to be the cause of this model mouse.

### The number of ribbon synapses in *Fgf22*^–/–^ mice kept intact

Our previous study suggested that FGF22 could preserve hearing by maintaining the number of ribbon synapses. The IHC ribbon synapses in *Fgf22*^–/–^ and *Fgf22*^+/+^ mice were clearly labeled by the double immunofluorescence staining. Antibodies against CtBP2 (red) and GluR2 (green) were used, respectively, to label presynaptic ribbons and postsynaptic glutamate receptors (Figure 3A). Juxtaposed puncta were identified as ribbon synapses and were quantified using 3D reconstruction. *Fgf22*^+/+^ and *Fgf22*^–/–^ mice did not significantly differ in their number of IHCs ribbon synapses (17.00 ± 0.8944 in *Fgf22*^+/+^ mice vs. 17.50 ± 1.376 in *Fgf22*^–/–^ mice; Figure 3B).

### Ribbon synapse synaptic vesicles was reduced in *Fgf22*^–/–^ mice

To quantify the number of vesicles per IHC ribbon synapse, sections of the IHCs from different *Fgf22*^–/–^ mice were imaged using TEM and subsequently compared with sections from *Fgf22*^+/+^ mice (*n* = 6 animals/genotype; Figure 4A). The number of ribbon SVs was significantly reduced in *Fgf22*^–/–^ mice than in *Fgf22*^+/+^ mice (11.40 ± 2.074 in *Fgf22*^–/–^ mice vs. 19.00 ± 1.581 in *Fgf22*^+/+^ mice; *P* < 0.01; Figure 4B).

### Alterations in Ca^2+^ current and exocytosis in *Fgf22*^–/–^ mice

To examine the role of FGF22 on ribbon synapses, we investigated the electrophysiological characteristics of Ca^2+^ currents using patch-clamp analysis (Figure 5A). The number of recorded IHCs was 15 in *Fgf 22*^+/+^ mice and 12 in *Fgf 22*^–^*^/^*^–^ mice. Neither the Ca^2+^ current amplitude (*I*_*Ca*_) nor the slope factor (*k*) of voltage-dependent calcium channels differed between *Fgf22*^–/–^ IHCs and those from wild-types (Figures 5B,C). A more negative half activation voltage (*V*_*half*_) was found in *Fgf22*^–/–^ IHCs, which indicated that the calcium channel was more easily activated in response to stimulus (Figure 5D).

To quantify exocytosis, a depolarizing pulse of 0 mV was applied to induce Ca^2+^ currents (Figure 6A). The stimulation durations ranged from 10 to 200 ms. During depolarization, we recorded capacitance changes (Δ*C*_*m*_) as well as changes in Ca^2+^ influx (*Q*_*Ca*_). For depolarization stimulations of 50, 100, and 200 ms, neither Δ*C*_*m*_ nor *Q*_*Ca*_ differed between *Fgf22*^–/–^ IHCs and those from wild-types. However, for depolarization stimulation durations of 10 and 30 ms, Δ*C*_*m*_ was significantly reduced in *Fgf22*^–/–^ IHCs, while no differences in *Q*_*Ca*_ were found between *Fgf22*^–/–^ and *Fgf22*^+/+^ IHCs (Figures 6B,C).

Afterward, we calculated the ratio of capacitance change to Ca^2+^ current charge (Δ*C*_*m*_/*Q*_*Ca*_) to quantify the efficiency of calcium-evoked exocytosis (Figure 6D). For depolarization stimulation durations of 10 and 100 ms, the Δ*C*_*m*_/*Q*_*Ca*_ in *FGF22*^–/–^ IHCs was significantly reduced compared to *Fgf22*^+/+^ IHCs. Meanwhile, Δ*C*_*m*_/*Q*_*Ca*_ was not altered for other stimulation durations (i.e., 30, 50, and 200 ms). These findings indicate that the decrease in Δ*C*_*m*_ for short stimuli results in the reduced efficiency of Ca^2+^-triggered exocytosis while Ca^2+^ currents remain.

### Modulation of SNAP-25, Gipc3, and MEF2D in *Fgf22*^–/–^ mice

SNAP-25 is essential for Ca^2+^-induced SV fusion and neurotransmitter release. Gipc3 (GAIP interacting protein, C terminus 3). Defects of the human GIPC3 gene cause human deafness and Gipc3 disruption in mice leads to audiogenic seizures and progressive hearing loss. MEF2D belongs to the myocyte-specific enhancer-binding factor 2 family and plays an important role in human development and physiological function. To investigate how FGF22 affected ribbon synapses, we used quantitative real-time PCR to compare mRNA expression levels of SNAP-25, Gipc3, and MEF2D between *Fgf22*^–/–^ and *Fgf22*^+/+^ mice. *Fgf22*^–/–^ mice displayed downregulation of SNAP-25 and Gipc3 and upregulation of MEF2D (Figure 7).

## Discussion

The main function of the IHC in the inner ear focuses on transducing sound waves into electric signals (Wang et al., 2017; Zhu et al., 2018; Qi et al., 2019, 2020; He et al., 2020). Deafness could be caused by genetic factors, infectious diseases, aging, ototoxic drugs, and noise exposure and is primarily attributed to the deterioration of cochlear hair cells (Zhu et al., 2018; Gao et al., 2019; Wagner and Shin, 2019; Zhang Y. et al., 2019; Qian et al., 2020; Zhang et al., 2020b; Zhou et al., 2020). Recently, much effort has been made to regenerate hair cells (White, 2020). Although the neonatal cochlea has limited hair cell regeneration ability, this regeneration ability decreases rapidly with increased age (Wang et al., 2015; Lu et al., 2017; He et al., 2019; Tan et al., 2019; Zhang S. et al., 2019; Zhang et al., 2020a,c). Many previous studies have reported that hair cell loss is mainly caused by oxidative damage (Liu et al., 2016; Li H. et al., 2018; Ding et al., 2020; Zhong et al., 2020; Zhou et al., 2020), which eventually induces apoptosis of hair cells (Sun et al., 2014; He et al., 2016; Yu X. et al., 2017; Li A. et al., 2018; Zhang Y. et al., 2019). Besides hair cells, the auditory nerve (AN) has been demonstrated to be involved in deafness, with pathogenic factors including noise, ototoxic drugs, and aging (Guo et al., 2016, 2019, 2020; Sun et al., 2016; Yan et al., 2018; Liu W. et al., 2019; Kohrman et al., 2020). Hearing dysfunction that cannot be detected by the hearing threshold is defined as HHL (Schaette and McAlpine, 2011). The relationship between ribbon synapses and FGF22 deletion remains indistinct. We previously demonstrated that FGF22 could be expressed in IHCs and gentamycin-induced hearing impairments occurred through ribbon synapse damage. Moreover, FGF22 infusion into the cochlea protected hearing by maintaining the number of ribbon synapses (Li et al., 2020). Here, we used 6-week-old mice with mature hearing.

We performed the ABR test in both *Fgf22*^–/–^ and *Fgf22*^+/+^ mice and found that the wave I amplitude was significantly decreased in *Fgf22*^–/–^ mice. The shift in wave I amplitude indicated impairment in sound-elicited discharges from the acoustic afferent nerve (Liu H. et al., 2019). However, wave I latency and hearing threshold were similar between the two groups of mice. The unaltered latency of wave I meant the unchanged traveling wave velocity in the basilar membrane of the cochlea, which may prove that the bundles of hair cells were unaffected (Popov and Supin, 2001), i.e., FGF22 deficiency-induced HHL. However, from the immunofluorescence staining, we found that the number of hair cells remained unchanged in *Fgf22*^–/–^ mice, as did the number of ribbon synapses.

Auditory signals are dynamically encoded at synapses and precisely transmitted from IHCs to SGNs (Wichmann and Moser, 2015). As part of the audio encoding, SVs containing neurotransmitters are released, which are continuously tethered at the active zones (Jeng et al., 2020). Vesicles are tightly coupled with calcium channels, which promote the rapid and sustained release of neurotransmitters. As the number of hair cells and ribbon synapses were kept intact, SVs were further studied and quantified using TEM. *Fgf22*^–^*^/^*^–^ IHCs exhibited reduced SVs, implying that FGF22 deficiency might led to a decline in vesicle formation. Besides, other explanations for such a decrease could be an impairment of vesicular replenishment, smaller ribbons, and a deficit in vesicular docking. We also assessed the biophysical characteristics of Ca^2+^ in IHCs. For basic properties of Ca^2+^ channels, a more negative half-activation voltage (*V*_*half*_) was presented in *Fgf22*^–/–^ mice, which revealed increasing excitability of Ca^2+^ channels in *Fgf22*^–/–^ mice. Considering the relevance between SVs and voltage-gated Ca^2+^ channels (VGCCs), we speculate that the conformation change triggers the rapid activation and deactivation of the Ca^2+^ channels, which influences the fusion of SVs (Atlas, 2013; Ghelani and Sigrist, 2018). Then, we applied a depolarizing pulse and different stimulus durations to measure calcium influx charge quantity (*Q*_*Ca*_), increments in membrane capacitance (Δ*C*_*m*_), and Δ*C*_*m*_/*Q*_*Ca*_. IHC depolarization triggered the fusion of vesicles with the cell membrane, which increased the cell membrane area and, accordingly, membrane capacitance. In *Fgf22*^–/–^ IHCs, the significantly decreased Δ*C*_*m*_ after short stimulation reflected the attenuated release of SVs. Due to unchanged *Q*_*Ca*_, the change in Δ*C*_*m*_ was not caused by differences in Ca^2+^ influx. Notably, Δ*C*_*m*_/*Q*_*Ca*_ was significantly decreased in *Fgf22*^–/–^ IHCs, which indicated that the reduction likely reflected a reduced efficiency in Ca^2+^-triggered exocytosis.

Altogether, we found the decreased wave I amplitude of ABR, the receding presynaptic SVs, the reduced Δ*C*_*m*_, and Δ*C*_*m*_/*Q*_*Ca*_, so we speculate that the reduction in neurotransmitter release by SVs was accompanied by weaker acoustic afferent fiber discharges and, accordingly, HHL.

To determine how *Fgf22* knockout reduces the amount of SVs, we performed real-time PCR to quantify the expression levels of SNAP-25 and Gipc3, important proteins for SVs. We found a significant decrease in SNAP-25 and Gipc3 mRNA levels. As a homologous protein of the SNAP protein family, SNAP-25 is essential for the fusion of Ca^2+^-induced SVs and neurotransmitter release (Najera et al., 2019). In SNAP-25 knockout neurons, rapid SV release is barely observed (Washbourne et al., 2002). Exocytosis at the hair cell ribbon synapse appears to function without neuronal SNARE proteins, according to Nouvian et al. (2011). However, we discovered that SNAP-25 and Gipc3 mRNA levels were significantly lower in *Fgf22*^–/–^ mice. Unknown mechanisms that are more sophisticated may exist. Previous studies have demonstrated that the Gipc3 allelomorph disrupts the configuration of the hair bundle (Charizopoulou et al., 2011). Moreover, Gipc3 mutations induce non-syndromic sensorineural hearing loss in humans (Katoh, 2013), and Gipc3 disruption enhances Ca^2+^ influx and exocytosis in IHCs, reverses the spatial gradient of maximal Ca^2+^ influx in IHCs, and increases the maximal firing rate of SGNs at sound onset (Ohn et al., 2016). While in this study, the decrease in Gipc3 expression was induced by the lack of Fgf22, it did not affect the Ca^2+^ current, only slightly shifting the Vhalf of the Ca^2+^ currents toward more hyperpolarized potentials and reducing exocytosis. We hypothesized that the absence of Fgf22 might have changed the expression of some proteins that affected calcium channel regulation, such as CtBP 2, though the expression level of calcium channel proteins was not thought to be impacted. The Gipc family may be involved in regulating vesicular trafficking (De Vries et al., 1998), and Gipc1 has been reported to be expressed in SVs at the presynaptic axon terminals of hippocampal neurons (Yano et al., 2006). Notably, we had reported that FGF22 triggered Ca^2+^ influx and activated calcineurin to downregulate MEF2D. Moreover, the transduction of cultured mouse cells with AAV-shFGF22 activated MEF2D and reduced the number of ribbon synapses (Li et al., 2020). MEF2D belongs to the myocyte-specific enhancer-binding factor 2 family and plays an important role in human development and physiological function. Here, we found that FGF22 knockout increased MEF2D expression and decreased the number of tethered SVs to the ribbon (Figure 4). Thus, we speculate that FGF signaling may antagonize the bone morphogenetic protein pathway to downregulate MEF2D (Cho et al., 2014). In addition, the myocyte enhancer factor 2 family may be associated with the WNT pathway (Snyder et al., 2013). These signaling pathways may regulate SV formation and release together. Regardless, the complex interplay between SNAP-25, Gipc3, and MEF2D needs to be further elucidated.

This study has some limitations. In this study, 6-week-old mice were used, and elder mice are under investigation. To sum up, despite this limitation, the mechanisms described above may offer novel therapeutic strategies to meet an ever-growing demand for deafness treatment.

## Conclusion

Our study lays the foundation for further elucidating the molecular mechanism of IHC ribbon synapses in HHL and provides a more in-depth understanding and potential clues to the pathophysiology of HHL from the regulatory genes. FGF22 deletion caused HHL by affecting the function of IHC ribbon synapses, which may offer a new idea and therapeutic target for hearing development in HHL.

## Data availability statement

The raw data supporting the conclusions of this article will be made available by the authors, without undue reservation.

## Ethics statement

The animal study was reviewed and approved by No. XHEC-F-2021-065. Written informed consent was obtained from the owners for the participation of their animals in this study.

## Author contributions

SH: study conception, data quality control, and wrote the manuscript. JZ: study conception, drafted the manuscript, and graphic abstract. YW: study conception, immunohistochemical staining, and drafted the manuscript. CJ: western blot and real-time PCR. HY: qPCR experiments. BH and YH: collection of cochlear samples for TEM experiments. YY: collection of cochlear samples for H&E experiments. JC: study conception. JY: data quality control and wrote the manuscript. SL: study conception, data quality control, and data analysis. All authors contributed to the article and approved the submitted version.

## Footnotes

1
www.bdbiosciences.com

2
www.millipore.com

## Abbreviations

IHCs: inner hair cells
SGNs: spiral ganglion neurons
HHL: hidden hearing loss
*Fgf22* ^–/–^: FGF22 knockout mice model
OHCs: outer hair cells
FGF: fibroblast growth factor
FGFR: fibroblast growth factor receptor
SNAP-25: synaptosome-associated protein 25
Gipc3: GIPC PDZ domain-containing family member 3
MEF2D: myocyte enhancer factor 2D
DNA: deoxyribonucleic acid
PCR: polymerase chain reaction
ABR: auditory brainstem response
SPLs: sound pressure levels
PFA: paraformaldehyde
CtBP2: C-terminal binding protein-2
GluR2: glutamate receptor 2
TEM: transmission electron microscopy
AN: auditory nerve
V_*half*_: half-activation voltage
VGCCs: voltage –gated Ca^2+^ channels
EDTA: ethylene diamine tetraacetic acid
SVs: synaptic vesicles.
