# Supplementary figures and images for: FGF22 deletion causes hidden hearing loss by affecting the function of inner hair cell ribbon synapses
Source: Front Mol Neurosci. 2022 Jul 28;15:922665. doi: 10.3389/fnmol.2022.922665 (PMC9366910; doi:10.3389/fnmol.2022.922665)

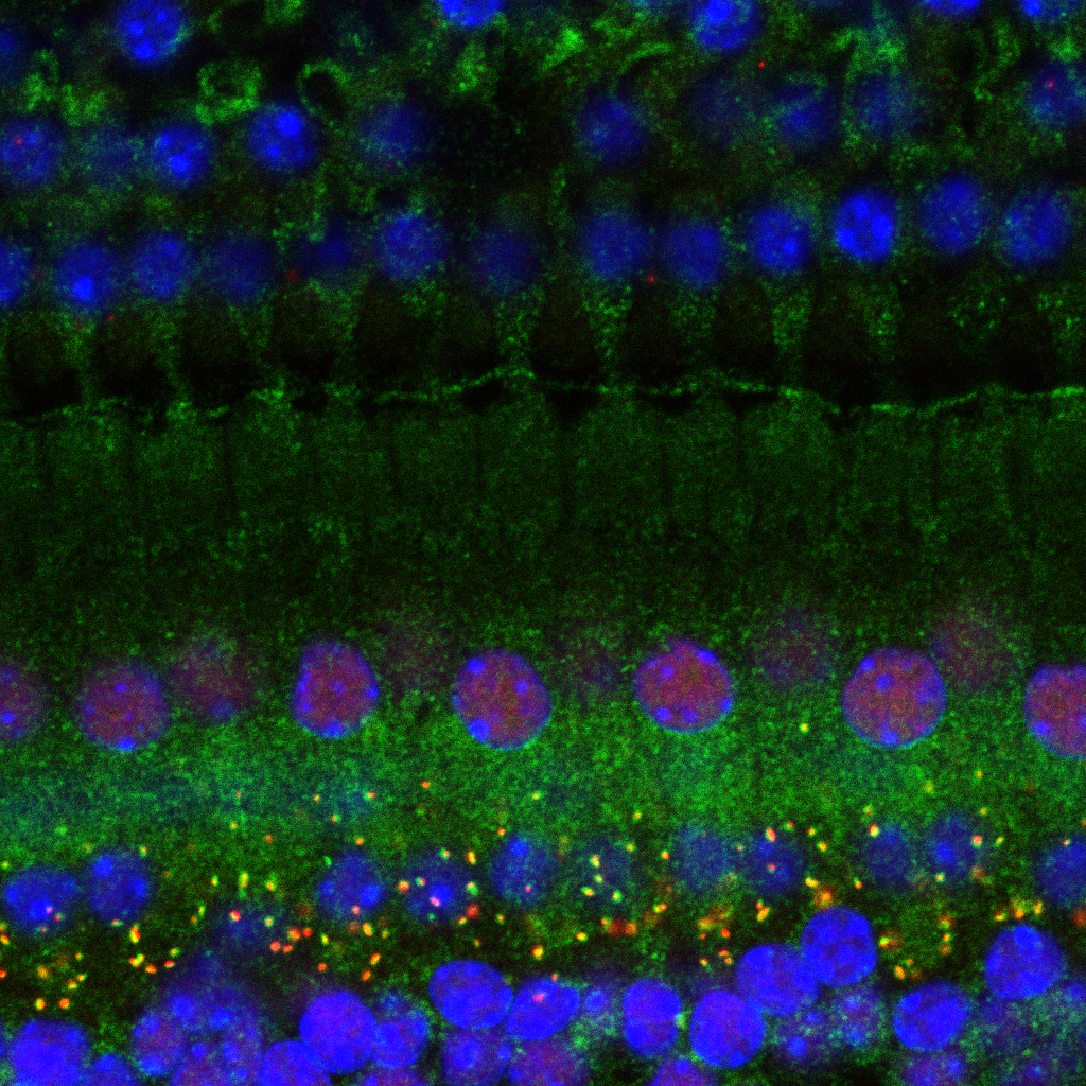

Supplement: Supplementary file 6 [file Data_Sheet_1.ZIP › confocal/20180508cph.lif_hom synapse-3Snapshot2.tif]

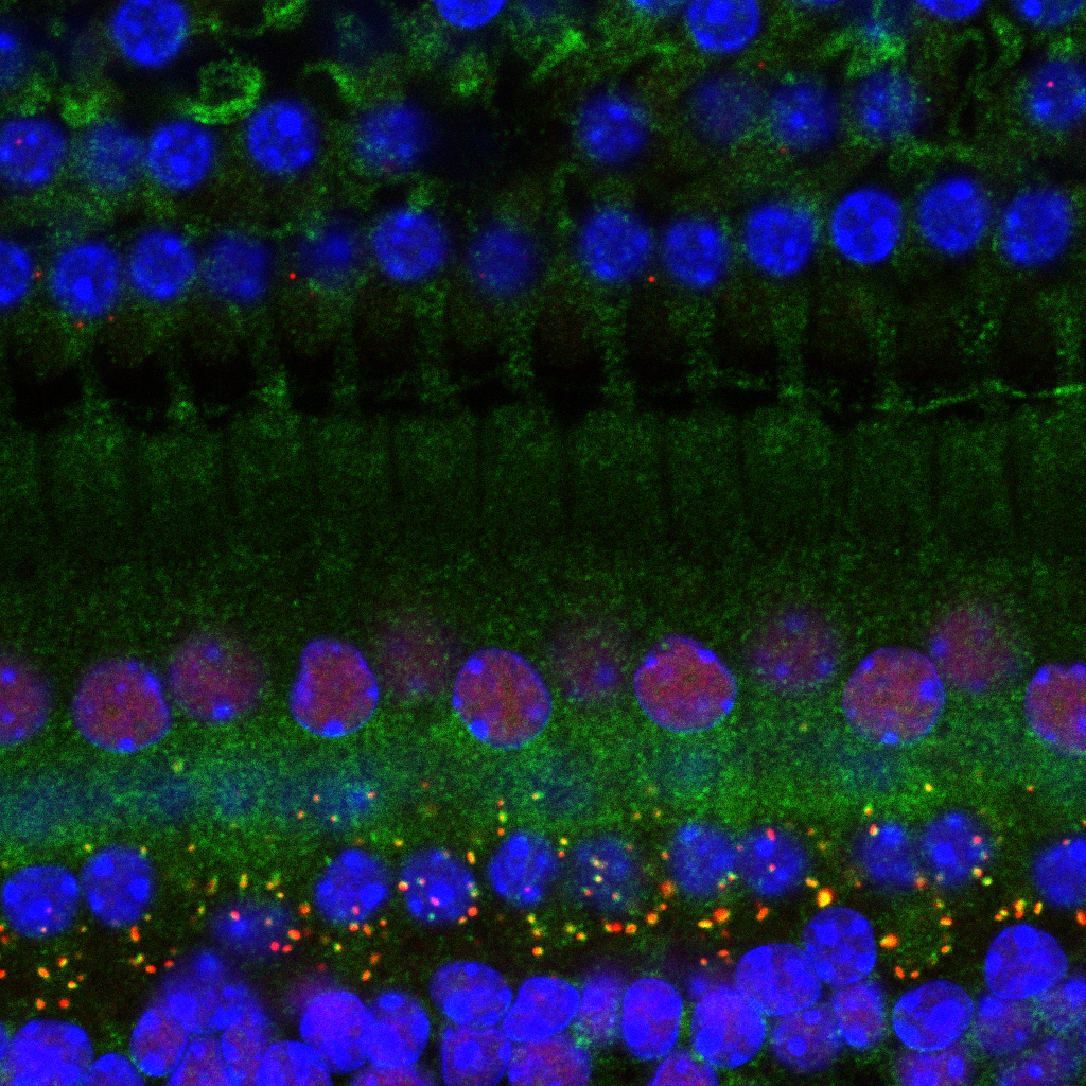

Supplement: Supplementary file 6 [file Data_Sheet_1.ZIP › confocal/20180508cph.lif_hom synapse-3Snapshot4.tif]

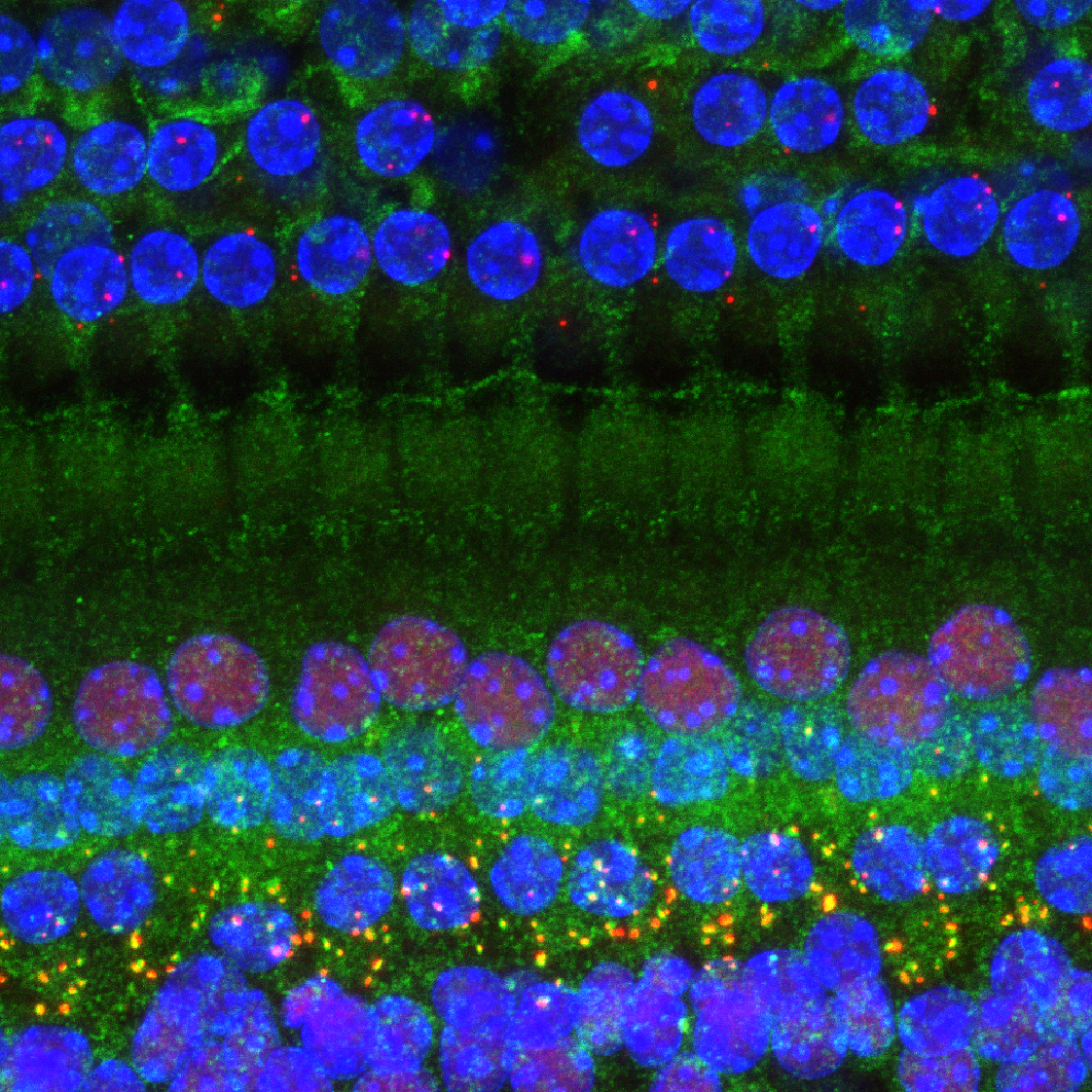

Supplement: Supplementary file 6 [file Data_Sheet_1.ZIP › confocal/20180508cph.lif_hom synapse-3Snapshot5.tif]

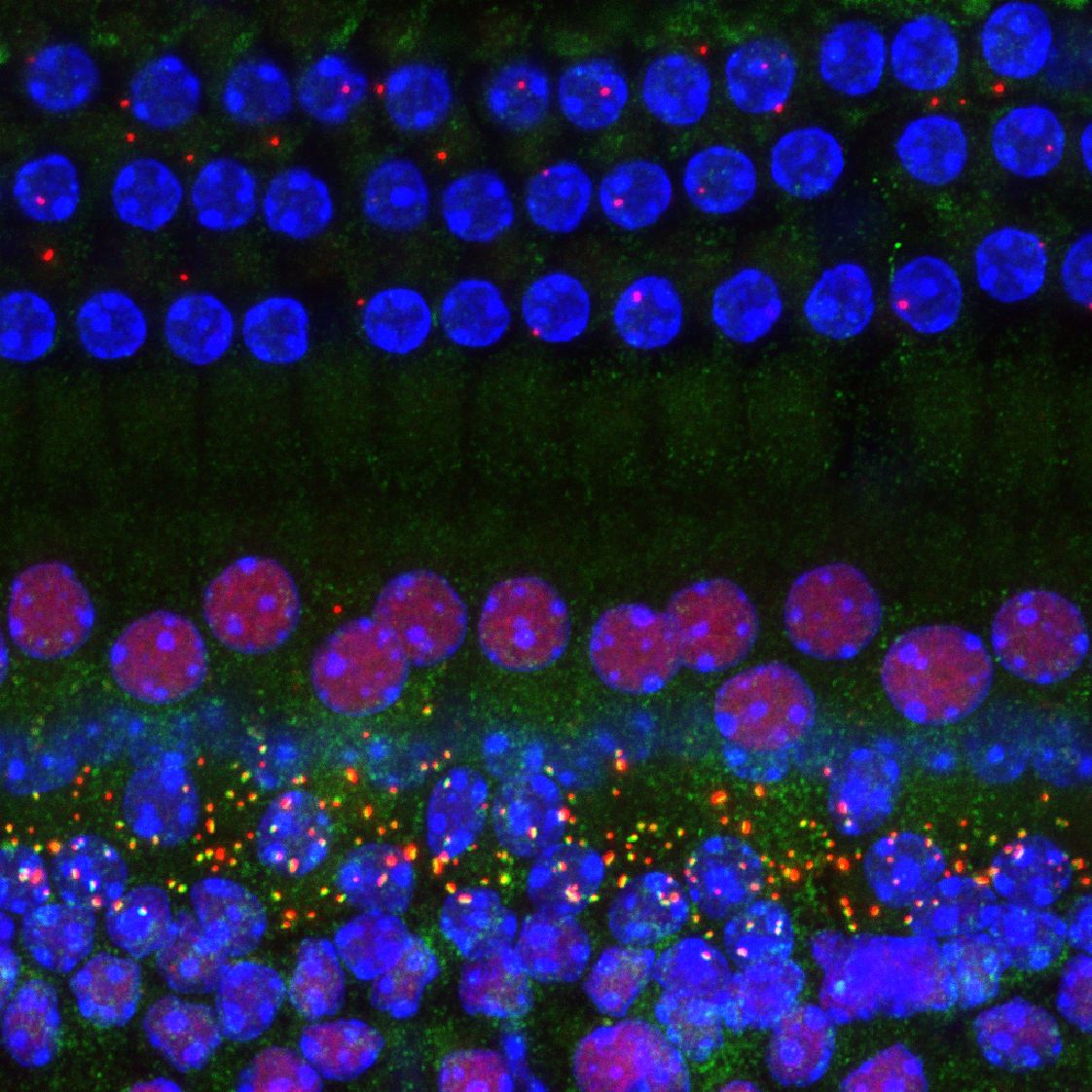

Supplement: Supplementary file 6 [file Data_Sheet_1.ZIP › confocal/20180508cph.lif_wt synapses-1Snapshot4.tif]

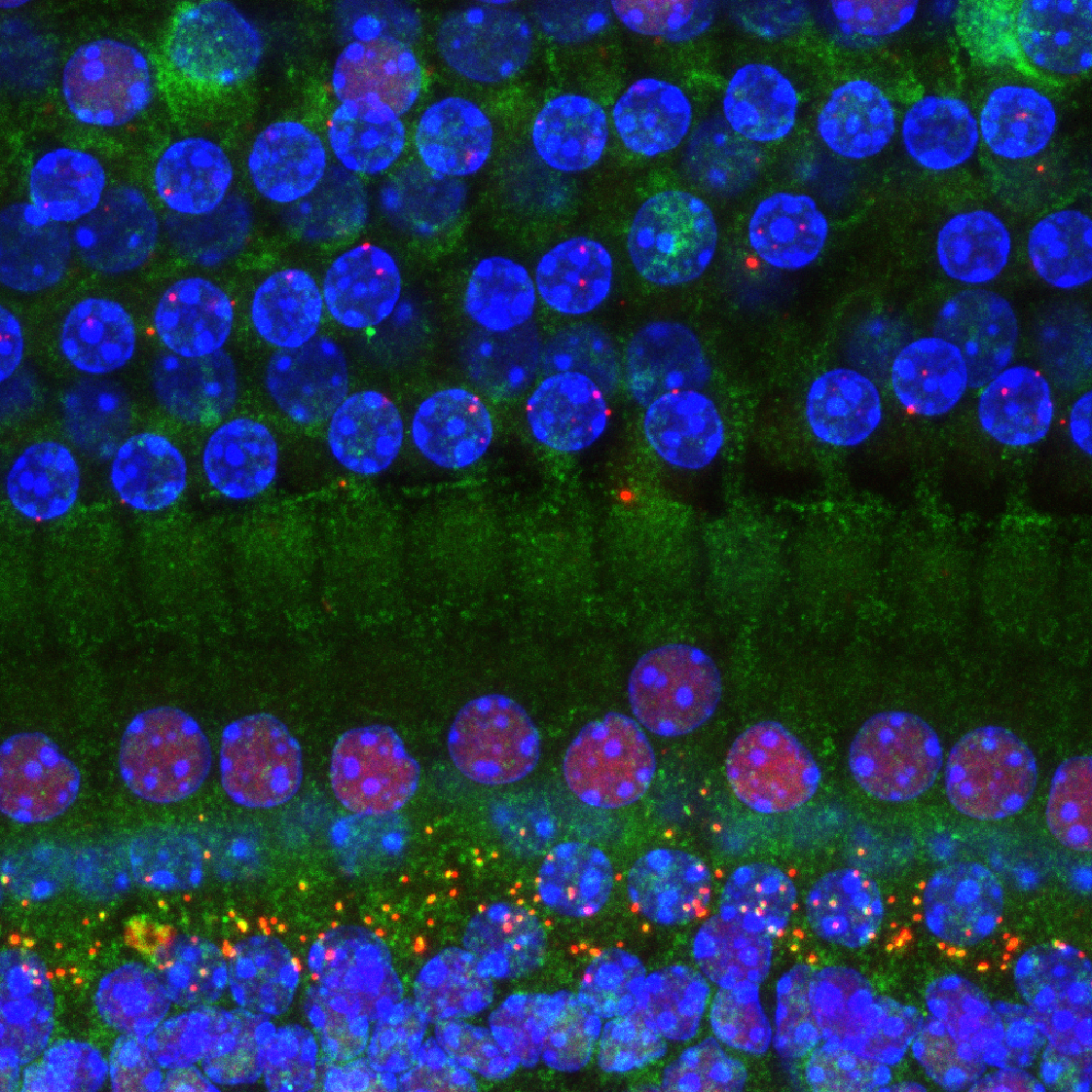

Supplement: Supplementary file 6 [file Data_Sheet_1.ZIP › confocal/20180508cph.lif_hom synapse-1Snapshot4.tif]

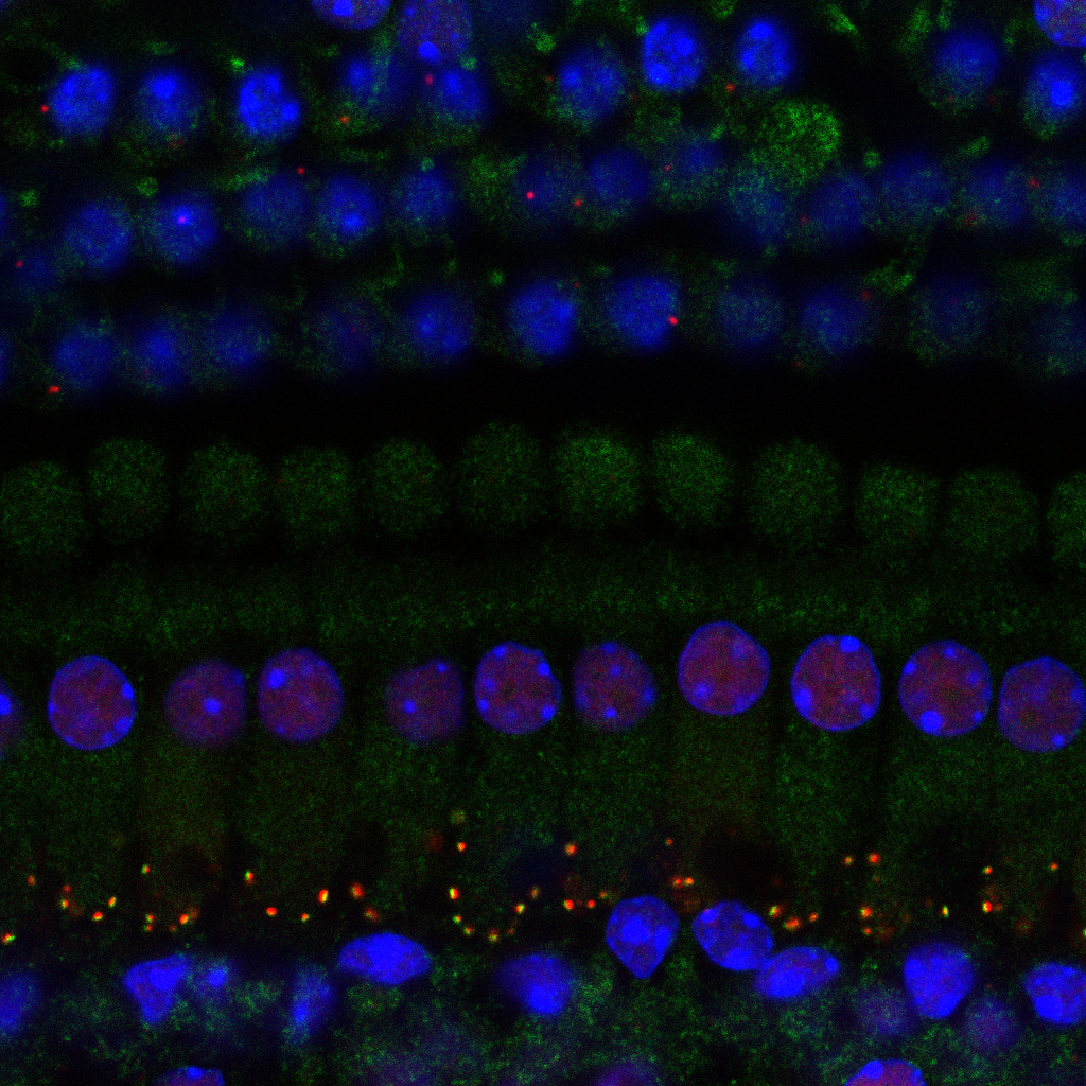

Supplement: Supplementary file 6 [file Data_Sheet_1.ZIP › confocal/20180508cph.lif_wt synapses -2Snapshot2.tif]

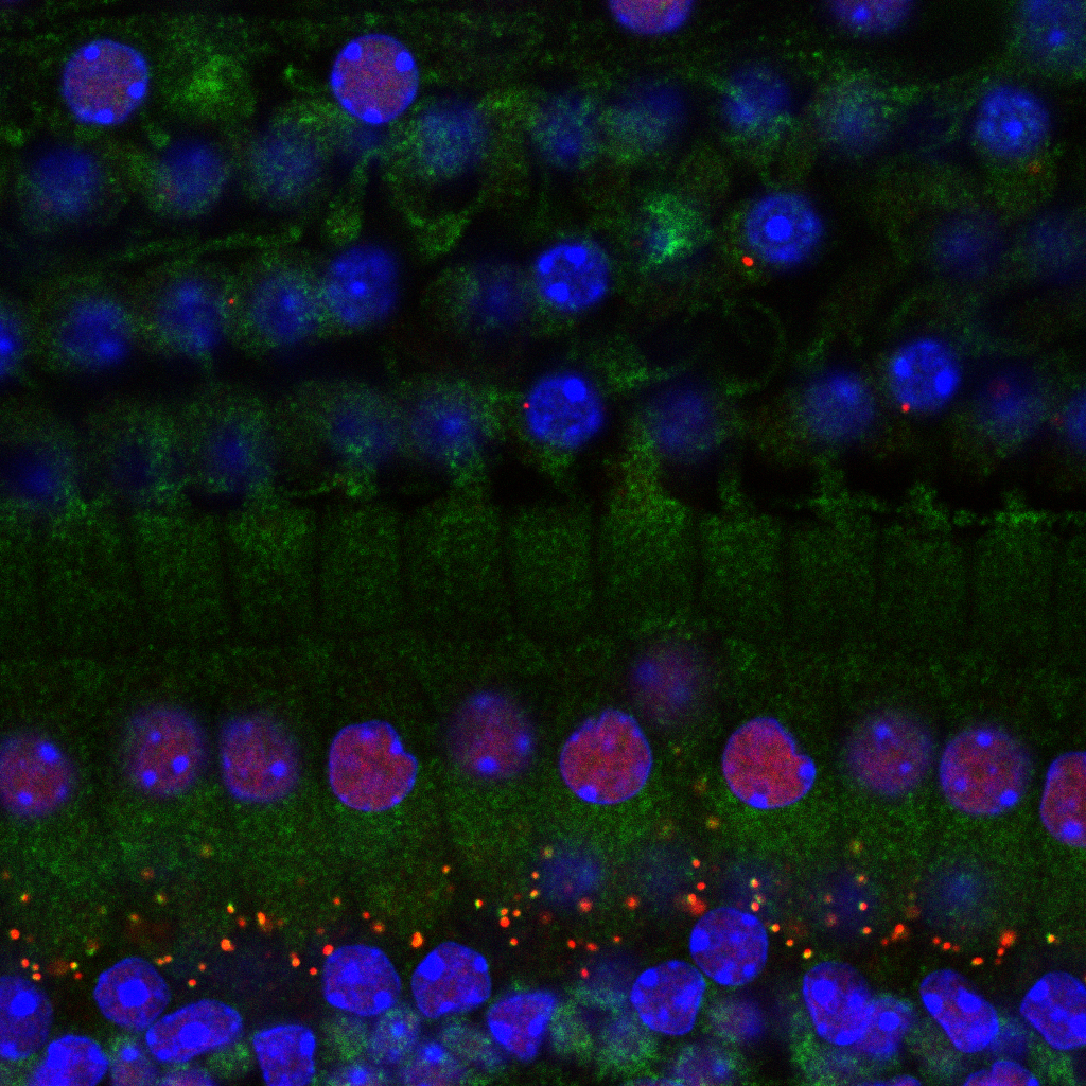

Supplement: Supplementary file 6 [file Data_Sheet_1.ZIP › confocal/20180508cph.lif_hom synapse-1Snapshot3.tif]

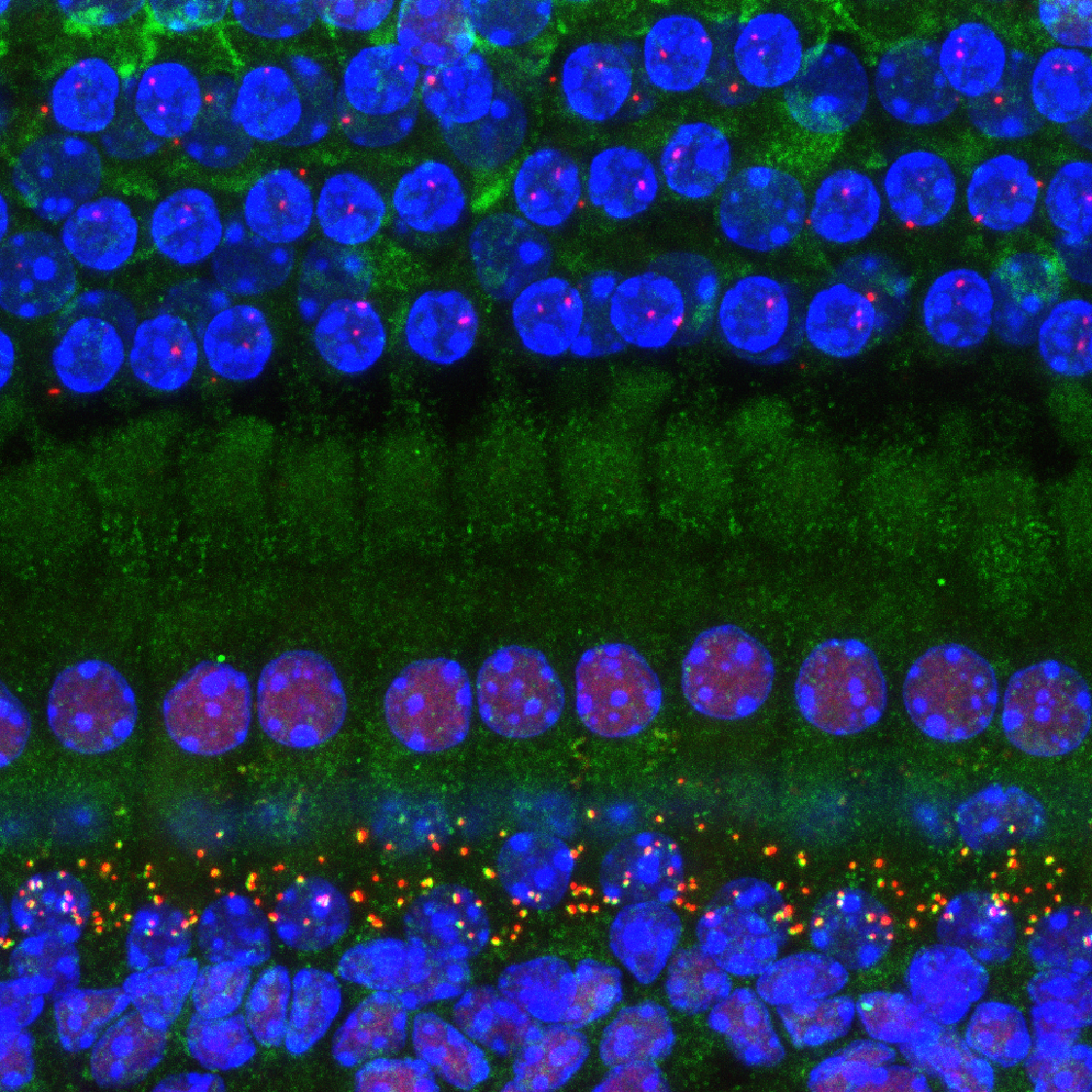

Supplement: Supplementary file 6 [file Data_Sheet_1.ZIP › confocal/20180508cph.lif_wt synapses -2Snapshot4.tif]

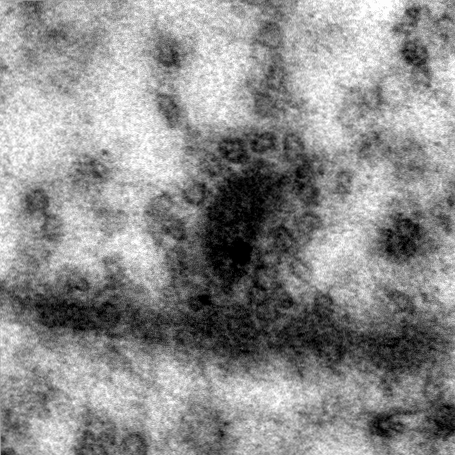

Supplement: Supplementary file 7 [file Data_Sheet_2.ZIP › Fig-TEM-ribbon/FGF22+:+0002.tif]
